# The Inhibition of Aluminum Corrosion in Sulfuric Acid by Poly(1-vinyl-3-alkyl-imidazolium Hexafluorophosphate)

**DOI:** 10.3390/ma7085711

**Published:** 2014-08-07

**Authors:** Paulina Arellanes-Lozada, Octavio Olivares-Xometl, Diego Guzmán-Lucero, Natalya V. Likhanova, Marco A. Domínguez-Aguilar, Irina V. Lijanova, Elsa Arce-Estrada

**Affiliations:** 1 Instituto Politécnico Nacional, ESIQIE, Departamento de Metalurgia y Materiales, Av. Instituto Politécnico Nacional S/N, Col. Lindavista, México D.F. 07300, Mexico; E-Mails: arellanessss@gmail.com (P.A.-L.); earsa@ipn.mx (E.A.-E.); 2 Facultad de Ingeniería Química, Benemérita Universidad Autónoma de Puebla, Av. San Claudio, Ciudad Universitaria. Col. San Manuel, Puebla, Puebla 72570, Mexico; 3 Instituto Mexicano del Petróleo, Programa de Investigación y Posgrado, Eje Central Lázaro Cárdenas No. 152, Col. San Bartolo Atepehuacán, México D.F. 07730, Mexico; E-Mails: djguzman@imp.mx (D.G.-L.); nvictoro@imp.mx (N.V.L.); madoming@imp.mx (M.A.D.-A.); 4 Instituto Politécnico Nacional, CIITEC, Cerrada Cecati S/N, Colonia Santa Catarina, Azcapotzalco, México D.F. 02250, Mexico; E-Mail: ivictorovnal@ipn.mx

**Keywords:** alloys, surfaces, corrosion test, electrochemical techniques, adsorption

## Abstract

Compounds of poly(ionic liquid)s (PILs), derived from imidazole with different alkylic chain lengths located in the third position of the imidazolium ring (poly(1-vinyl-3-dodecyl-imidazolium) (PImC_12_), poly(1-vinyl-3-octylimidazolium) (PImC_8_) and poly(1-vinyl-3-butylimidazolium) (PImC_4_) hexafluorophosphate) were synthesized. These compounds were tested as corrosion inhibitors on aluminum alloy AA6061 in diluted sulfuric acid (0.1–1 M H_2_SO_4_) by weight loss tests, polarization resistance measurements and inductively coupled plasma optical emission spectroscopy. Langmuir’s isotherms suggested film formation on bare alloy while standard free energy indicated inhibition by a physisorption process. However, compound efficiencies as inhibitors ranked low (PImC_12_ > PImC_8_ > PImC_4_) to reach 61% for PImC_12_ in highly diluted acidic solution. Apparently, the high mobility of sulfates favored their adsorption in comparison to PILs. The surface film displayed general corrosion, and pitting occurred as a consequence of PILs’ partial inhibition along with a continuous dissolution of defective patchy film on formation. A slight improvement in efficiency was displayed by compounds having high molecular weight and a long alkyl chain, as a consequence of steric hindrance and PIL interactions.

## 1. Introduction

Aluminum and its alloys find a wide variety of technological applications owing to the balanced combination of physical, mechanical and chemical properties, such as electrical and thermal conductivities, low density, high ductility, stability for surface treatments, good corrosion resistance, easiness of recycling and functional advantages of extruded and cast semi-products [1,2,3]. The addition of elements, such as copper, lithium, manganese, magnesium, silicon and zinc, allows aluminum to achieve a long range of bulk properties; likewise, minor additions provide an improvement in specific properties [4].

Aluminum generally exhibits passive behavior in aqueous solution due to the formation of a strong and compact adherent passive oxide film on the surface, which affects corrosion susceptibility. The adhesive passivating surface oxide film is amphoteric, and consequently, the metal is readily dissolved when exposed to aggressive acidic and alkaline solutions. In fact, the corrosion of metallic materials in acidic solution causes considerable costs [5,6]. In near-neutral solutions, the solubility of oxides will be much lower than in acidic solutions, and the extent of dissolution will tend to be smaller [7,8].

The presence of aggressive ions (chlorides, fluorides) attacks the oxide film locally, and certain elements (Ga, Tl, In, Sn, Pb, Fe) are incorporated into it, which may have a deleterious effect. Most commercial alloys contain several types of intermetallic phases, where aluminum corrosion occurred essentially as a micro-galvanic process between these secondary phases and the matrix alloy [9]. Sulfuric acid solutions are widely used for aluminum anodizing operations. Additionally, aluminum and its alloys are designated for industrial applications to manage a large variety of acid and alkaline solutions, but once dissolved, corrosion of the naturally-formed film can only be controlled by efficient inhibitors.

The use of chemicals as corrosion inhibitors (CIs) is one of the most popular and efficient methods to protect metals; this is a convenient and relatively inexpensive method to achieve this goal [10,11,12,13]. Organic compounds are commonly used as CIs in industry, such as those having π electrons in structure and containing nitrogen, sulfur, phosphorus and oxygen atoms, through which they are adsorbed on the metal surface [14,15,16,17,18,19]. The adsorption is influenced by the electronic structure of the inhibiting molecules [20] and also by steric factors, aromaticity, electron density at the donor atoms and the presence of functional groups [21,22,23]. The use of polymers as CIs has attracted considerable attention due to their inherent stability and cost effectiveness [24,25,26,27,28]. Several families of homopolymers have been widely studied: polyacrylamide, polyvinyl pyrrolidone, polyacrylic acid and polyethyleneimine, It is known that polymers are strongly adsorbed on the surface, though giving lower toxicity than their analogues; hence, it is expected that polymers will be better corrosion inhibitors than the corresponding monomers [29,30]. Polymers may provide some advantages in comparison to other organic molecules: They provide a large single chain that displaces water molecules from metallic surface and makes inhibition more favorable. The presence of multiple bonding sites on the metallic surface makes polymer desorption a slow process [31]. Polymers form complexes with metal ions, and these complexes occupy a larger surface area, thereby blanketing the surface and protecting metal from the corrosive agents contained in solution [32].

In the present work, the effect of three poly(ionic liquid)s (PILs) (poly(1-vinyl-3-dodecylimidazolium hexafluorophosphate), poly(1-vinyl-3-octylimidazolium hexafluorophosphate) and poly(1-vinyl-3-butylimidazolium hexafluorophosphate)) were tested as CIs in a concentration range within 10 to 100 mg·L^−1^ (ppm) in aqueous sulfuric acid to prevent the corrosion of aluminum alloy AA6061. Weight loss tests, potentiodynamic polarization and surface analysis were used to support the proposed mechanism of inhibition. As not many polymers are considered as good corrosion inhibitors for non-ferrous metals and its alloys since surface adsorption is not always a straightforward process, it was decided to synthesize polymers based on ILs, as they possess a polymeric structure with surfactant components having positive and negative charges along with a hydrophobic chain

## 2. Experimental Section

### 2.1. Synthesis and Characterization of PILs

PILs were synthesized and characterized by ^1^H and ^13^C NMR and IR spectroscopies. Melting points were measured in a Fisher Scientific apparatus equipped with a 300 °C thermometer. FT-IR spectra were registered on a Nicolet FT-IR 5DX FT spectrophotometer as KBr discs. ^1^H NMR (300 MHz) and ^13^C NMR (75.4 MHz) spectra were obtained in a JEOL Eclipse-300 equipment using TMS as the internal standard. DMSO-d6 was used as solvent for PImC_8_ and PImC_4_, and CDCl_3_ for PImC_12_, in each case, dissolution was performed at room temperature. The gel permeation chromatography system was performed in a Waters 2410 refractive index detector with a 3 PL gel 10-μm mixed-B columns to determine molecular weights. The GPC was calibrated with a polystyrene standard with tetrahydrofuran as the eluent.

The monomers of 1-vinyl-3-alkylimidazolium hexafluorophosphate were obtained according to the reported procedure [33], while the polymerization of 1-vinyl-3-alkylimidazolium hexafluorophosphates was carried out by free radical polymerization in solution using 2,2ʹ-azo-bis(isobutyronitrile) as described elsewhere [34] to obtain white solid products in a 97% yield. Table 1 shows the common chemical name and abbreviation for the compounds used in the present study. The polymerization was proven by the disappearance of the peaks corresponding to the vinyl group in the ^1^H NMR at 5.44, 5.96 and 7.26 ppm, ^13^C NMR at 108.6 and 119.1 ppm [19] and the appearance of new peaks corresponding to the polyvinyl group at around 3.24 and 2.2 ppm in ^1^H NMR (Table 2).

**Table 1:** The poly(ionic liquid)s (PILs) tested as corrosion inhibitors.

Abbreviation	Name	Structure	MW (g/mol)	DP	IR, cm^−1^
**PImC_12_**	Poly(1-vinyl-3-dodecylimidazolium hexafluorophosphate)	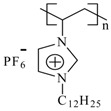	60,500	148	3,168, 2,934, 2,879, 1,553, 1,475, 837, 738, 555
**PImC_8_**	Poly(1-vinyl-3-octylimidazolium hexafluorophosphate)	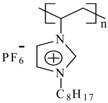	51,400	145	3,168, 2,931, 2,859, 1,554, 1,469, 1,164, 835, 736, 557
**PImC_4_**	Poly(1-vinyl-3-butylimidazolium hexafluorophosphate)	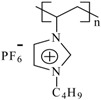	32,300	108	3,166, 2,969, 2,881, 1,552, 1,471, 1,162, 836, 738, 557

**Table 2:** NMR characterization of synthesized PILs.

IL	^1^H NMR, ppm				^13^C NMR, ppm		
	Alkylic chain	Imidazolium ring	Polymeric chain	N-CH_2_	Alkylic chain	Imidazolium ring	N-CH_2_
PImC_12_	0.89 (b, 3H) 1.26 (b, 18H) 1.72 (b, 2H)	7.32 (b, 2H) 8.35 (b, 1H)	1.86 (b, 2H) 2.95 (b, 1H)	4.09 b, 2H	14.30, 22.91, 26.51, 29.17 (2C), 29.60 (2C), 29.90 (3C), 32.16	123.83, 129.42, 136.12	51.60
PImC_8_	0.89 (b, 3H) 1.29 (b, 10H) 1.69 (b, 2H)	7.89 (b, 2H) 8.62 (b, 1H)	2.28 (b, 2H) 3.24 (t, 1H)	3.94 b, 2H	13.57, 21.78, 25.70, 28.15, 28.41, 28.89, 30.95	123.83, 128.87, 136.12	49.52
PImC_4_	0.96 (b, 3H) 1.35 (b, 2H) 1.70 (b, 2H)	7.68 (b, 1H) 7.89 (b, 1H) 8.66 (b, 1H)	2.27 (b, 2H) 3.24 (b, 1H)	3.97 b, 2H	12.88, 18.78, 30.63	123.44, 129.12, 134.49	49.08

### 2.2. Materials Preparation

The experiments were performed on alloy AA6061 samples, because it is widely used in different technological applications. Its chemical composition is as follows (wt%): 0.4/0.8 Si, 0.7 Fe, 0.15/0.4 Cu, 0.15 Mn, 0.8/1.2 Mg, 0.04/0.35 Cr, 0.25 Zn, 0.15 Ti, balanced with Al. Specimens were abraded with wet SiC paper number 400–4,000, degreased in hexane and washed in an ultrasonic bath of acetone for 5 min to remove impurities. The contact area used in the polarization resistance tests was 0.196 cm^2^; in order to do so, cylindrical specimens of 1.0 cm × 0.5 cm were mounted in epoxy resin to secure the contact area.

### 2.3. Test Solution

The test solution was prepared by using sulfuric acid of standard grade and deionized water. Aqueous solutions of H_2_SO_4_ (0.1, 0.3, 0.7 and 1.0 M) were used to test PILs as CIs for AA6061. The compounds were added to the acid solutions in concentrations of 10, 30, 75 and 100 mg·L^−1^ (ppm).

### 2.4. Weight Loss Measurements

The cylindrical specimens (1.5 cm × 0.5 cm) for weight loss measurements were immersed in solutions of diluted sulfuric acid with and without PILs (100 mg·L^−1^). Inductively coupled plasma mass spectrometer (ICP-OES) model Varian 730-ES was employed to determine the amount of aluminum ions in H_2_SO_4_ solutions by the EPA 6010C method [35], after the immersion of aluminum alloy in the test solution for 30 days.

### 2.5. Electrochemical Test

Electrochemical measurements were carried out in a standard three-electrode cell. The counter electrode was a cylindrical bar of graphite; the reference electrode consisted of a commercial saturated calomel electrode (SCE) with a Luggin capillary probe, and the working electrode made of aluminum alloy AA6061 was embedded into a cylindrical bar made of Teflon. Tests were performed in a naturally aerated solution kept at a temperature of 25 ± 1 °C. Electrochemical tests were performed in a potentiostat/galvanostat PGSTAT302N controlled by a PC through the general purpose electrochemical system (GPES). Before recording the polarization resistance measurements (*R*_p_), the working electrode was immersed in the test solution until the steady-state open circuit potential (*E*_OCP_) was reached (~15 min). Polarization scans were then performed at a rate scan of 0.166 mV s^−1^ in the potential range of ±20 mV *vs. E*_OCP_.

### 2.6. Surface Analysis

AA6061 aluminum alloy coupons were exposed to 1.0 M H_2_SO_4_ in the absence and presence of PILs (100 mg·L^−1^) from 3 h to 30 days at 25 ± 1 °C. The metallic surface condition was recorded by SEM (scanning electron microscope) model JEOL-JSM-6300. Results of semi-quantitative elemental composition on sample were obtained by an EDX (electron dispersive X-ray) analyzer module attached to the microscope.

## 3. Results and Discussion

### 3.1. Weight Loss Tests

The corrosion rate of aluminum alloy AA6061 in the diluted solutions of H_2_SO_4_ was determined through the ICP-OES technique in the presence and absence of PILs after sample immersion for 30 days. Remainder solutions rich in aluminum ions provided the concentration of metallic ions in mg·L^−1^ after applying an analytical procedure [35]. From these concentrations and initial solution volume, the amount of aluminum ions in grams was determined. These values were expressed as weight loss measurements (W) to determine the corrosion rate by the following Equation [36].


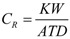
(1)
where *C_R_* is the corrosion rate (mm/year), *K* is a constant (8.76 × 10^4^), *W* is the weight loss (g), *T* is the time the sample was exposed to the corrosive environment (h), *A* is the surface area of exposed metallic surface (cm^2^) and *D* is the metal density (g/cm^3^).

**Figure 1:**
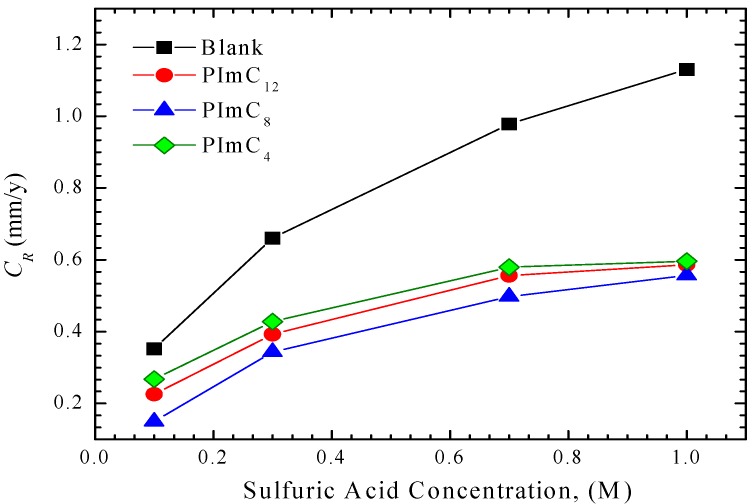
Corrosion rate of aluminum alloy AA6061 in acidic solutions at 25 °C measured by the ICP-OES method.

Figure 1 shows the results obtained from gravimetric tests aided by the ICP-OES technique. It is observed that *C*_R_ increased with the concentration of sulfuric acid in water, in which the pH was determined (1.1, 0.9, 0.6 and 0.5) for the different sulfuric acid concentrations (0.1, 0.3, 0.7 and 1.0) as a measure of fluid aggressiveness.

As higher concentrations of sulfates implied a higher concentrations of Al^3+^ ions in the aqueous environment, the reduction reactions on the cathode are catalyzed, which, in turn, accelerates oxidation reactions, thereby producing an increase in the corrosion rate. Figure 1 shows that the presence of PILs decreased the corrosion rate as inhibitor molecules were adsorbed on the aluminum surface to form a protective film that worked as a barrier that partially prevented ionic interchange; this way, the kinetics of electrochemical reactions are slowed and the corrosion rate on the metal surface is mitigated.

### 3.2. Electrochemical Test

**Figure 2:**
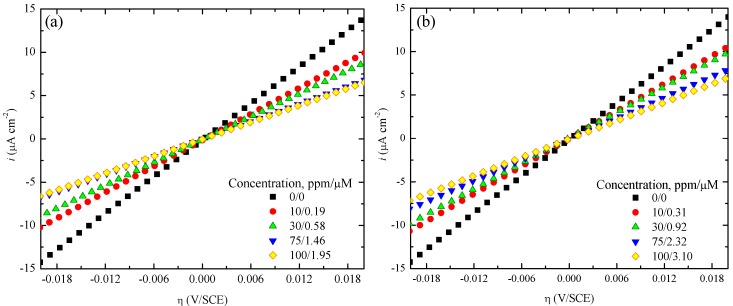
Potentiodynamic polarization curves of aluminum alloy AA6061 in 0.1 M H_2_SO_4_ with (**a**) PImC_8_ and (**b**) PImC_4_.

Figure 2a,b show the graphs of current density (*i*) as a function of overpotential (η) for AA6061 in 0.1 M H_2_SO_4_ with and without the presence of PImC_8_ and PImC_4_, respectively. Similar curves to those displayed were obtained for the different inhibitor and acid concentrations. Slopes from figures show a decay as the PIL concentration is increased in the aqueous solution. In agreement with theory, *R*_p_ is inversely proportional to the potential current curve slope, which means that an increase in the concentration of CI produces an increase in the polarization resistance, and as a result, the corrosion rate is decreased. Similar curves to those displayed were obtained for the different inhibitor concentrations and acidic solutions.

Table 3 summarizes the polarization resistances (*R*_p_) for the tested concentrations in acid with and without CIs. The results in absence of PILs were 1423, 969, 940 and 924 Ω cm^2^ for the testing solution of H_2_SO_4_ at 0.1, 0.3, 0.7 and 1.0 M, respectively. An increase in the concentration of H_2_SO_4_ resulted in a decrease of the *R*_p_ values, which implied an increase in metal dissolution; this observation was also supported by the ICP-OES tests that suggested an increase of the metallic ions in solution as shown in Figure 1. *R*_p_ tended to be proportional to PIL concentration; this behavior is derived from an increase in the resistance of the metal solution interface due to the film formed by the inhibitor and corrosion products that together contributed to isolating the metallic surface from ionic interchange, which led to surface passivation and contributed to mitigating the corrosion rate of alloy AA6061.

*R*_p_ values (Table 3) followed the order: PImC_12_ > PImC_8_ > PImC_4_. It is observed that the alkylic chain had an impact on the polarization resistance and, therefore, on corrosion inhibition, as reported elsewhere [37]; this way, PImC_12_ formed a denser film on the surface to enable greater protection when compared to the aliphatic chain lengths of the other PILs. Likewise, it is thought that the average molecular mass of polymer PImC_12_ (60,500 g/mol, *DP* = 148) and PImC_8_ (51,400 g/mol, *DP* = 145) may contribute to the increase of *R*_p_ due to the more efficient arrangement of polymeric film on the metallic surface. In contrast, PImC_4_ (32,300 g/mol, *DP* = 108) is less efficient as a result of both a smaller backbone and a smaller size of alkylic side chain than the others. Both factors contributed synergistically to decrease the metal-solution interaction with the consequent increase in *R*_p_ and the decrease in the corrosion rate.

Table 3 shows the change of *E*_corr_ in the presence and absence of PIL compounds as a function of sulfuric acid molarity. These numbers display the maximum difference in absolute value between the corrosion potential of the blank material and any combination of PIL concentration and acid molarity; as a result, we have the largest differences, which are useful in determining the inhibitory nature. It is observed that the corrosion potentials in the anodic branches of PImC_12_, PImC_8_ and PImC_4_ are 17, 25 and 41 mV (*vs*. SCE); whereas for the cathodic branches, they are 25, 25 and 45 mV (*vs*. SCE), respectively. As none of these values surpassed the reference change of 85 mV in any direction, it is claimed that PILs can be classified as mixed type inhibitors [38].

**Table 3:** Electrochemical parameters derived from polarization resistance tests on aluminum alloy AA6061 at 25 °C. CI, corrosion inhibitor.

CI	Concentration (ppm/µM)	0.1 M H_2_SO_4_		0.3 M H_2_SO_4_		0.7 M H_2_SO_4_		1.0 M H_2_SO_4_	
		*R*_p_ (Ohm·cm^2^)	*−E*_corr_ (mV)	*R*_p_ (Ω·cm^2^)	*−E*_corr_ (mV)	*R*_p_ (Ω·cm^2^)	*−E*_corr_ (mV)	*R*_p_ (Ω·cm^2^)	*−E*_corr_ (mV)
PImC_12_	Blank materialAA6061	1,423	657	969	651	940	621	924	653
	10/0.16	2,270	658	1,340	647	1,288	639	970	636
	30/0.49	2,523	653	1,491	676	1,373	620	1,072	642
	75/1.24	3,334	650	1,952	660	1,543	644	1,203	659
	100/1.65	3,663	641	2,070	658	1,667	639	1,242	654
PImC_8_	10/0.19	1,982	670	1,313	646	1,245	643	930	652
	30/0.58	2,260	647	1,405	655	1,345	646	1,013	650
	75/1.46	2,942	646	1,843	642	1,469	641	1,131	655
	100/1.95	3,052	642	2,003	639	1,543	644	1,176	659
PImC_4_	10/0.31	1,889	657	1,283	669	1,240	666	929	618
	30/0.92	2,034	663	1,369	665	1,308	632	1,001	612
	75/2.32	2,495	646	1,679	665	1,434	643	1,074	623
	100/3.10	2,832	647	1,727	655	1,517	655	1,136	623

The corrosion inhibitor efficiency *I*_E_ (%) of PILs was determined by the *R*_p_ values:

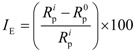
(2)
where *R*_p_*^i^* and *R*_p_^0^ are the corresponding measurements determined in the presence and absence of PILs. In Table 4, a summary of the *I*_E_ is provided for each PIL concentration as a function of the sulfuric acid molarity. The values suggested that PILs mitigated corrosion on the testing material AA6061. It is important to note that the top efficiency was obtained at 100 mg·L^−1^ for every PIL under test, even though PImC_12_ with an alkylic chain composed of 12 carbon atoms displayed the highest *I*_E_ (~61%).

A possible explanation of the relatively fair inhibitor performance may include the following: A film of aluminum oxide (Al_2_O_3_) is spontaneously formed on the alloy; this film provides protection until it is partially dissolved in the acidic solution [39]. However, on inhibition treatment, the PIL formed another film on the alloy [40], which is partially compact and defective; a lack of compactness is derived from deficient chain stacking, which avoids forming a fully dense polymeric film on the substrate. Furthermore, the relatively high concentrations of sulfuric acid and low ones of the inhibitor in solution implied a large amount of sulfate ions and free protons; both chemical species compete with PILs macromolecules in occupying the active sites on the metallic surface. A chance to create defects at the interface metal-film due to hydrogen accumulation and sulfate ions’ high mobility is likely, although in acid corrosion, the anodic reactions predominate, as metal is lost through the dissolution of sulfates and blistering is not generated. Additionally, the patchy nature of the protective film of the inhibitor allowed for local activity; these conditions contributed to secondary oxidation reactions, which prevented the surface from having complete coverage and a higher PIL efficiency. This process occurred and had a deleterious effect on the corrosion inhibitory efficiency despite the high molecular weight (backbone) and alkylic side chain) and long backbone of the PIL molecule.

**Table 4:** Inhibition efficiency of PILs on aluminum alloy at 25 °C.

CI	Concentration (ppm/µM)	*I*_E_ (%)			
		0.1 M H_2_SO_4_	0.3 M H_2_SO_4_	0.7 M H_2_SO_4_	1.0 M H_2_SO_4_
PImC_12_	10/0.16	37	28	27	5
	30/0.49	44	35	31	14
	75/1.24	57	50	39	23
	100/1.65	61	53	44	25
PImC_8_	10/0.19	28	26	24	1
	30/0.58	37	31	30	9
	75/1.46	52	47	36	18
	100/1.95	53	52	39	21
PImC_4_	10/0.31	25	24	24	1
	30/0.92	30	29	28	8
	75/2.32	43	42	34	14
	100/3.10	50	44	38	19

In Table 4 is observed that PILs efficiency depends on their concentration in solution, and for each concentration, there is a particular time to reach their highest efficiency. Likewise it was observed that PImC_12_ reached the highest efficiency despite the similar structure to another PIL, but it has the largest aliphatic side chain; these parameters suggest that efficiency depends on the molecular structure of inhibitor, inhibitor concentration, aggressiveness of testing environment and type of metallic material. However, these parameters are integrated in the nature of the adsorption process, as the efficiency of organic molecules depends on their capability of being adsorbed on the metal surface. It is worth noting that adsorption on the corroded surfaces never reaches the real equilibrium, but it tends to a steady state; when corrosion is sufficiently small, adsorption tends to be in quasi-equilibrium [41], but in contrast, when corrosion is sufficiently high, adsorption is difficult to reach, which is the case of sulfates over PIL molecules. This way, every PIL required a certain time and concentration for a specific structure to reach its peak efficiency.

**Figure 3:**
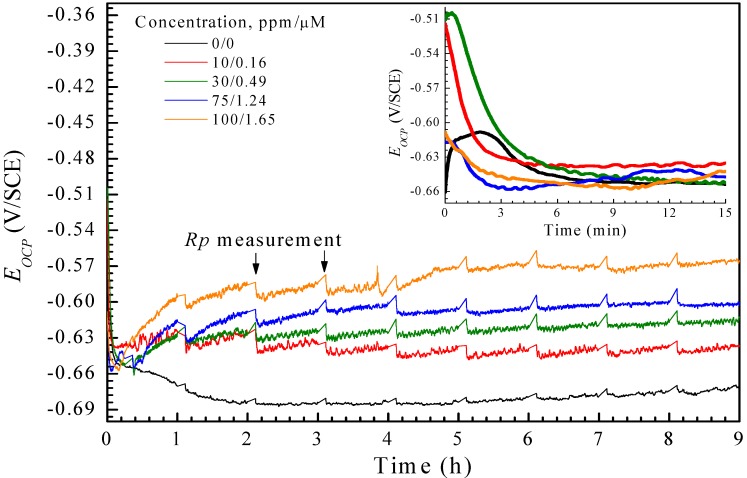
Time dependence of open circuit potential of aluminum alloy AA6061 in 1.0 M H_2_SO_4_ acquired in the presence of PImC_12_.

The open circuit potential as a function of time was determined in the testing solutions in absence of electric polarization to have an insight of PIL adsorption on the alloy AA 6061 under naturally aerated conditions. Figure 3 displays the corresponding measurements of *E*_OCP_ (*vs*. SCE) as a function of immersion time at 1.0 M H_2_SO_4_ in the presence of PImC_12_. In the absence of a corrosion inhibitor, for the first minutes, the curve potential time displayed a displacement towards positive values due to the rapid formation of the passive film of aluminum oxide as a result of its highly active nature. For longer times, in the range of one hour, the curve was displaced to negative values of potential; this tendency was ascribed to the dissolution of the oxide passive film as a result of the adsorption of sulfate ions (SO_4_^2−^) and the formation of subsequent soluble complex Al_2_(SO_4_)_3_(H_2_O)*_n_*. The cathodic reactions involved in hydrogen evolution on the aluminum surface contributed with the SO_4_^2−^ ions to the damage of the passive film. After two hours of potential monitoring, the *E*_OCP_ tended to equilibrium at about −685 mV (*vs*. SCE), which was ascribed to the corrosion potential *E*_corr_ [42].

The addition of PImC_12_ to the acidic solution showed a drastic change in the behavior of the *E*_OCP_ in comparison to the solution in the absence of the inhibitor. At the beginning, a displacement to negative potential values was observed due to the dissolution of passive film, whereas at higher concentrations of PImC_12_, within a time period of 30 minutes, *E*_OCP_ showed an increase to positive values, now due to film formation with molecules of H_2_O, SO_4_^2−^, aluminum oxides and PImC_12_, which contributed to corrosion protection [43]. In summary, the addition of PImC_12_ to the system moved *E*_corr_ to positive potentials; a similar behavior was observed for PImC_8_ and PImC_4_. The anodic displacements of *E*_corr_ for PImC_12_, PImC_8_ and PImC_4_ were about 100 mV, 80 mV and 70 mV *vs.* SCE, respectively, which suggested that the presence of CIs affected the aggressive environment and mainly the anodic reaction within the testing times.

Polarization scans were performed following the potential measurements to determine the film formation stability. The curve profile in Figure 3 displays the decay of *E*_OCP_ every hour, and Table 5 provides the values of *I*_E_ obtained from *R*_p_ of the alloy with and without CIs as immersion time dependent. When immersion time was increased, the PILs efficiency decreased, apparently because film formation tended to be less compact as a result of macromolecular desorption, which provided an increase of the active sites occupied by H_2_, SO_4_^2−^, OH^−^ and H_3_O^+^. The *R*_p_ values as a function of testing time and concentration followed the same order: PImC_12_ > PImC_8_ > PImC_4_, as indicated by previous results.

**Table 5:** Inhibition efficiency of PILs on aluminum alloy AA6061 as a function of immersion times at 25 °C.

CI	Time (h)	*I*_E_ (%)			
		10 ppm	30 ppm	75 ppm	100 ppm
PImC_12_	1	18	23	40	63
	3	24	30	37	66
	6	23	30	36	55
	9	21	29	38	53
PImC_8_	1	15	20	38	59
	3	18	21	30	61
	6	20	26	29	47
	9	18	21	30	47
PImC_4_	1	14	16	26	44
	3	12	19	27	39
	6	15	20	25	41
	9	10	15	30	41

### 3.3. Adsorption Isotherms

The effect of inhibition on the corrosion of metallic materials is mainly ascribed to the molecular adsorption of the corrosion inhibitor on the metal surface. A metallic surface when in contact with an aqueous solution is saturated with water molecules, whereas in the presence of a corrosion inhibitor, these molecules displaced the adsorbed water. On water substitution by CI molecules, the latter block the active sites on the metallic surface where corrosion reactions occur.

Isotherms provide an idea of the adsorption and desorption processes and the interactions of metal-inhibitor molecules on the metallic surface. To understand the inhibition process of AA6061 in sulfuric acid, the experimental data were related to adsorption isotherms, which described the operative process between inhibitor-metal. Langmuir (Equation (3)), Temkin (Equation (4)) and Freundlich (Equation (5)) were considered to observe PILs tendency [44]:

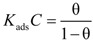
(3)

*K*_ads_*C* = e^*f*θ^ (4)

*K*_ads_ = θ (5)

where *K*_ads_ is the equilibrium constant of adsorption, *C* is the inhibitor concentration, *f* is the interaction constant and θ the fraction of the surface covered; the latter parameter has a direct relation with the inhibitor efficiency by means of the following equation [45].


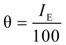
(6)

**Figure 4:**
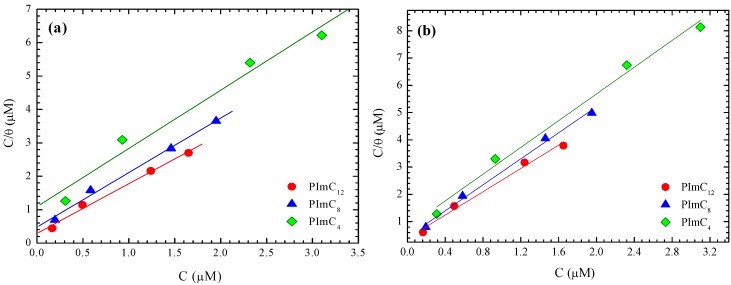
Langmuir’s isotherms for aluminum alloy AA6061 in (**a**) 0.1 M and (**b**) 0.7 M H_2_SO_4_ solutions with PILs as CIs.

Figure 4a,b shows the fitting of experimental data based on Langmuir’s adsorption model when testing A6061 in 0.1 M and 0.7 M H_2_SO_4_, respectively. In Table 6, it is observed that the correlation coefficients were close to the unit, which corroborated that PILs are relatively easily adsorbed on the metal surface and follow the predicted model of the Langmuir isotherm.

**Table 6:** Summary of fundamental adsorption constants of PILs of aluminum alloy AA6061 at 25 °C.

H_2_SO_4_ Solution (M)	CI	*R*^2^	Slope	*K*_ads_ (mmol^−1^)	−Δ*G*^0^_ads_ (kJ·mol^−1^)
0.1	PImC_12_	0.98	1.49	3,431	30.1
	PImC_8_	0.98	1.63	2,089	28.9
	PImC_4_	0.95	1.74	917	26.9
0.3	PImC_12_	0.98	1.63	2,219	29.1
	PImC_8_	0.96	1.65	1,550	28.2
	PImC_4_	0.98	1.99	1,062	27.2
0.7	PImC_12_	0.98	2.14	2,567	29.4
	PImC_8_	0.99	2.38	2,253	29.1
	PImC_4_	0.98	2.45	1,276	27.7
1.0	PImC_12_	0.99	2.09	350	25.0
	PImC_8_	0.97	1.73	179	22.8
	PImC_4_	0.75	2.21	96	21.3

**Figure 5:**
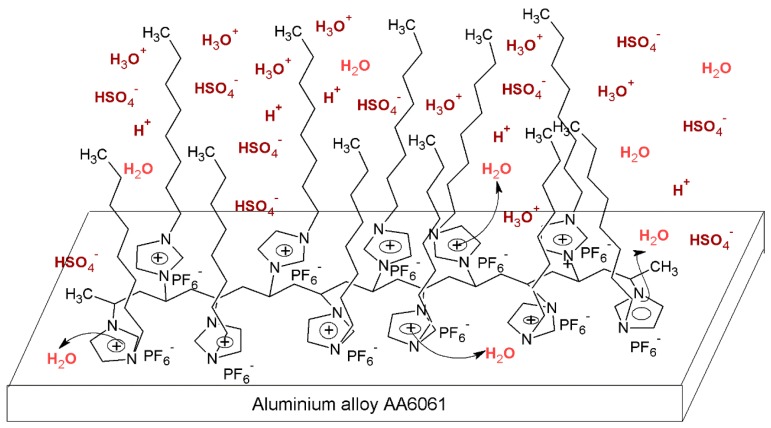
Schematic representation of molecular adsorption and later PIL inhibition of aluminum alloy AA6061 in diluted sulfuric acid.

The values of *K*_ads_ obtained from the intersection of Langmuir’s isotherms to the origin are reported in Table 6 for the presence of PImC_12_; the highest values of *K*_ads_ were obtained for the different concentrations tested in diluted sulfuric acid. This indicated that the compounds of high molecular weights (PImC_12_) and long side chains were more easily adsorbed on the alloy surface. It appeared that the alkyl side groups of each repeating unit of the polymeric CIs naturally created hydrophobic interactions among them, which decreased available water molecules in content to interact with other hydrophobic tails. This way, an alkyl side group of longer aliphatic chains present in the backbone can produce stronger hydrophobic interactions, which, in turn, decreased the amount of aggressive ions affecting the metallic surface [46]. Figure 5 provides an idea of PILs’ interaction with both the metallic surface and the aggressive environment in an attempt to describe a likely mechanism of adsorption and later PIL inhibition of AA6061 in diluted sulfuric acid. The interaction among the hydrophobic parts of the PILs molecules could support the protective action. However, it is more likely that the main chains of the polymer form an obstacle, as the lateral alkylic chains were haphazardly placed along the main chains of the polymer, which may have a supportive action on inhibition, as they hinder the pass of water and aggressive ions, in agreement with their hydrophobic nature. The steric hindrance is more efficiently carried out when the alkyl side chain is composed of 12 carbons, as it can interact with the other lateral alkylic groups to slow molecular diffusion. The probability of these interactions among alkylic side chains consisting of eight and four carbons decreased markedly, so that water molecules can pass more easily. The main chain of the macromolecules can be placed one above the other as a result of the opposite ionic attractions that can evolve in different points of two or more adjacent macrochains to form the protective film.

The values of *K*_ads_ were used to determine the standard free energy of adsorption (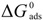) as indicated in the following Equation:

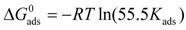
(7)
where *R* is the gas universal constant (8.314 J·mol^−1^·K^−1^), T is the absolute temperature of the aggressive medium, (*K*_ads_) and 55.5 stands for the molar concentration of water (M). The values of 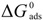 are displayed in Table 6, which showed only negative ones, suggesting that the Cis’ adsorption process occurred, that is to say, PILs formed an adsorbed film on the aluminum alloy surface. PILs in the aqueous media produced a negative change in the adsorption free energy as a result of water and ion displacement from the metallic surface. Negative values persisted even at 0.1 M H_2_SO_4_ as an indication that PILs adsorption is favored even when the number of sulfate aggressive ions and protons in solution tended to be lower.

When the values of 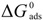 are equal to −20 kJ·mol^−1^ or less negative, this thermodynamic parameter suggests an adsorption process determined by the electrostatic attraction forces between the ionic charges and the dipoles of the adsorbed chemical species and the electric charge of metal in the metal-solution interface, which indicates that a physisorption process has occurred [47]. On the other hand, the 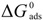 derived in values of −40 kJ·mol^−1^ or more negative ones suggested that a chemisorption process is under control, in which the CIs are capable of forming chemical bonds with the surface as electrons move into the metallic surface to form a coordinated type of bond with the metallic surface [48]. The values of 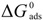 for these molecules were determined in the range of −30 to −21 kJ·mol^−1^, which means that compounds were adsorbed on the metallic surface by a process of physical adsorption.

### 3.4. Surface Analysis

#### 3.4.1. Sample Surface after 3 h of Immersion

**Figure 6:**
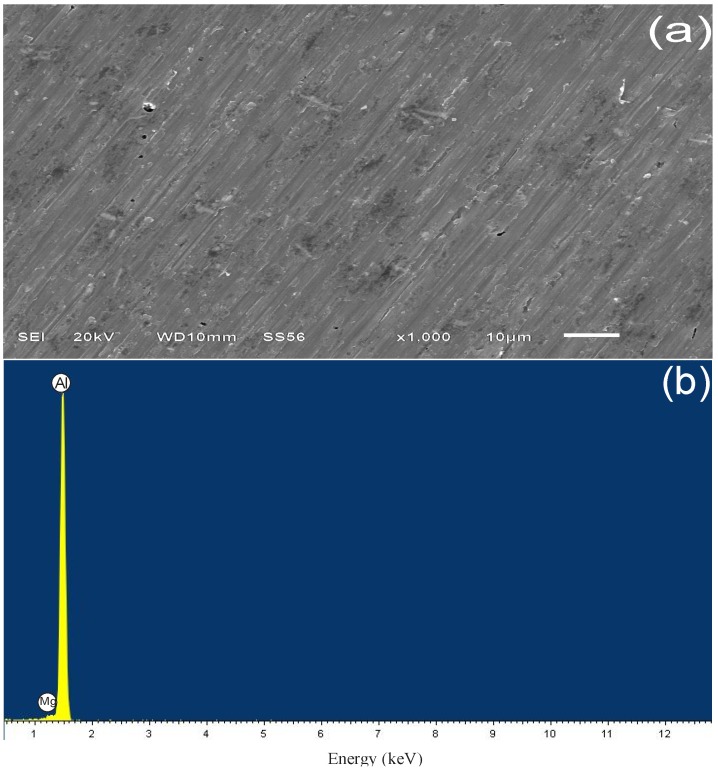
(**a**) SEM and (**b**) EDX analysis of aluminum alloy AA6061 before exposure to the acidic medium.

**Figure 7:**
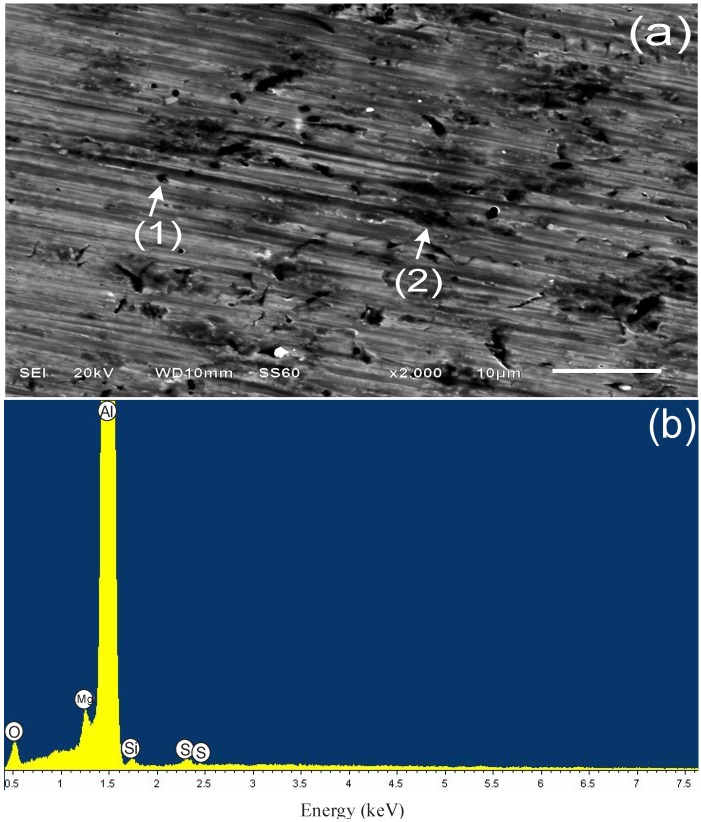
SEM (**a**) and EDX (**b**) analysis of aluminum alloy AA6061 after exposure for 3 h in 1.0 M H_2_SO_4_.

Figure 6a shows the scanning electron micrograph of alloy AA6061 without etching, where a surface is observed with oriented and uniform lines produced by mechanical polishing; EDX analysis in Figure 6b indicated that aluminum is the main constituent in the alloy. Figure 7a shows the surface appearance of the alloy after immersion in 1.0 M H_2_SO_4_ for three hours, where a heterogeneous surface with small pits of different form (1,2) is displayed. In this case, the EDX spectrum described the characteristic signals of aluminum, in addition to other elements, such as oxygen and sulfur, which are common as corrosion products of aluminum in sulfuric acid when a complex of type Al_2_(SO_4_)_3_(H_2_O)*_n_* is formed [49].

**Figure 8:**
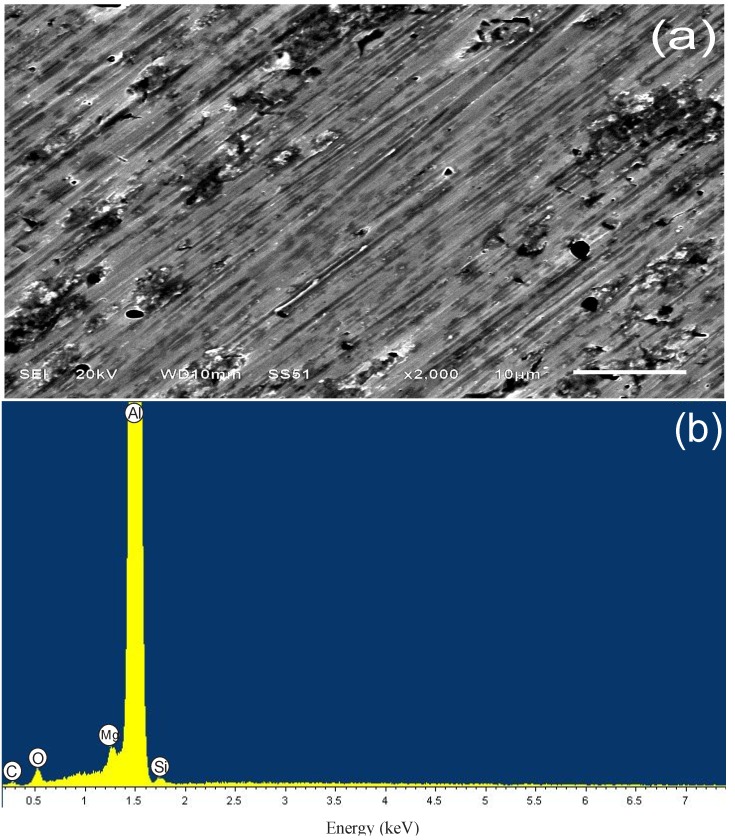
(**a**) SEM and (**b**) EDX analysis of aluminum alloy AA6061 after exposure for three hours in 1.0 M H_2_SO_4_ with the addition of 100 ppm of PImC_12_.

The micrograph in Figure 8a shows the alloy surface in the presence of PImC_12_. In comparison to the blank morphology, there are regions displaying general corrosion in addition to pits with an average lower density and individual diameters. EDX analysis in Figure 8b corroborated the presence of oxygen in a lower concentration, and sulfur appeared in the spectrum, which suggested that PILs decreased the formation of the soluble complex Al_2_(SO_4_)_3_(H_2_O)*_n_* and, thereby, decreased the kinetics of the corrosion reactions. The carbon signal, which was absent in the blank material, was attributed to the presence of PILs molecules on the surface; the presence of PImC_12_, PImC_8_, and PImC_4_ qualitatively indicated a carbon content of 4.8% to roughly 32% in different regions of the alloy surface. Table 7 shows the percentages of the elements present on the analyzed surfaces with and without IC presence.

**Table 7:** Weight percent of the elements present in the AA6061 alloy after a 3-h exposure in 1.0 M H_2_SO_4_.

Spectrum	Weight %						
	C	O	Mg	Al	Si	S	Total
Without attack	–	–	1.02	98.98	–	–	100
Blank	–	6.17	0.9	91.83	0.64	0.46	100
PImC_12_	8.28	5.01	0.76	85.3	0.66	–	100
PImC_8_	4.83	2.77	0.78	91.12	0.51	–	100
PImC_4_	9.42	3.52	0.74	85.67	0.65	–	100

#### 3.4.2. Sample Surface after 30 Days of Immersion

**Figure 9:**
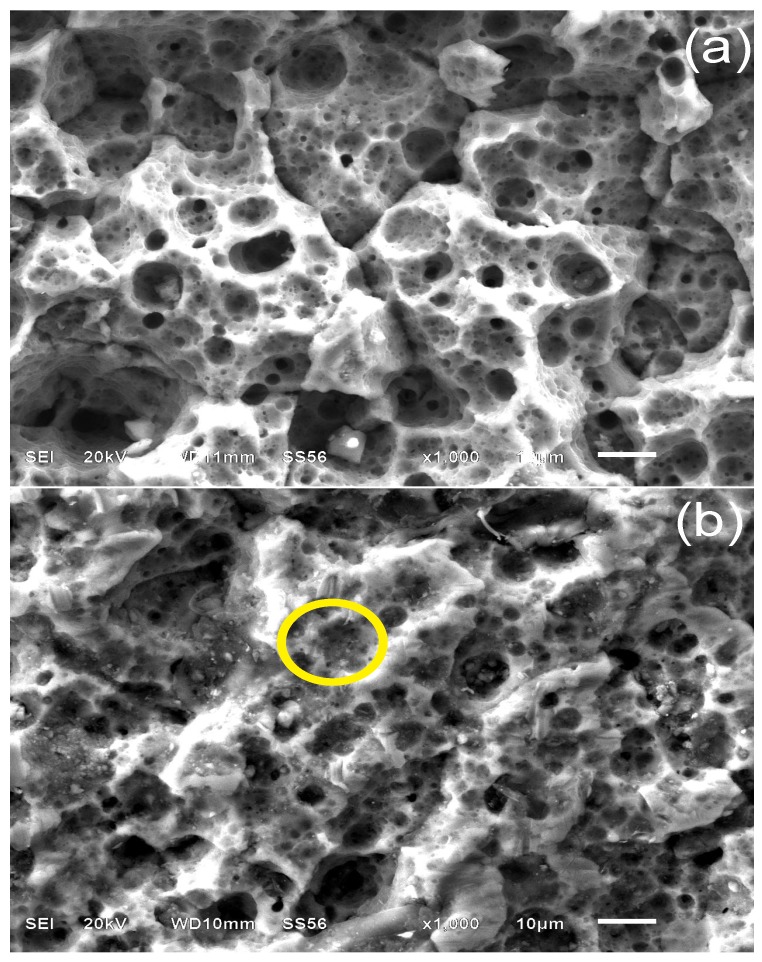
SEM of aluminum alloy AA6061 surface after exposure for 30 days in acidic solution (**a**) without PIL and (**b**) with 100 mg·L^−1^ of PImC_12_.

Figure 9a shows the micrograph of the AA6061 surface after an immersion time of 30 days in 1.0 M H_2_SO_4_ without CI. A rearrangement of porous corrosion products in islands is observed as a result of the limited growth. Porosity allows a continuous diffusion of aggressive ions and, as a result, the continuous corrosion of metal. On addition of 100 ppm (1.65 × 10^−3^ mM) of PImC_12_ in acidified solution (Figure 9), the alloy surface displayed the following important changes: (1) the boundaries among the corrosion products are not distinguished; and (2) the number and diameter of holes decreased. The EDX analysis of the zone highlighted in yellow indicated the presence of carbon (34.2 wt%) in a higher concentration when compared to the analysis referred to in Figure 8b, which suggests that the adsorption process is relatively continuous, probably because of the affinity that could exist between the complex Al_2_(SO_4_^2−^)_3_(H_2_O)*_n_* and the inhibitor. Additionally, the CI can also be present on the corrosion products, and since chemical interactions could not be necessarily on the active sites, the PILs’ efficiency as inhibitors was adversely affected.

**Figure 10:**
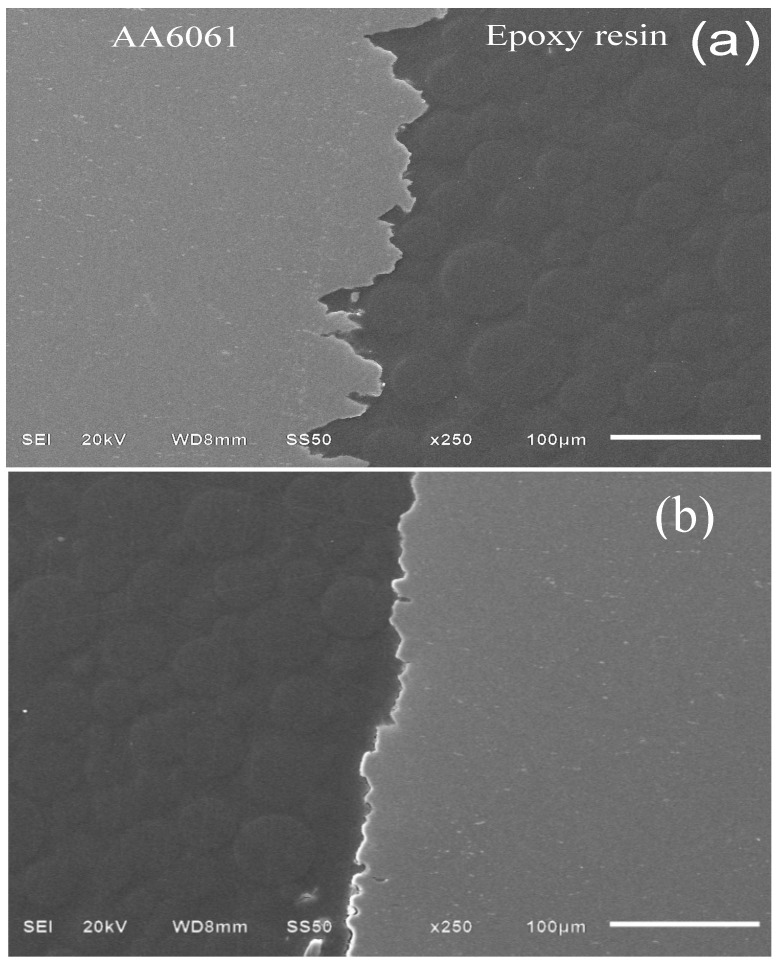
SEM image of a cross-section of the aluminum alloy AA6061 surface after exposure for 30 days in the acidic solution (**a**) without PILs and (**b**) with 100 mg·L^−1^ of PImC_12_.

Figure 10 shows a cross-section micrograph of alloy AA6061 after testing for 30 days. Pitting is associated with (a) Al_2_(SO_4_^2−^)_3_(H_2_O)*_n_*, as the surface revealed pits with a depth of approximately 50 µm. Figure 10b corresponds to a micrograph with the addition of PImC_12_. In comparison to Figure 10a, pit type is mainly elliptic and depth decreased around 15 µm. The decrease in pit depth and their change in pit morphology indicated that PIL protected metal surface and, therefore, decreased this type of localized damage.

### 3.5. Adsorption Mechanism

The inhibitor efficiency of PILs against corrosion of alloy AA6061 in aqueous solution of H_2_SO_4_ is a result of molecular adsorption at the metal-solution interface. As a natural reaction of aluminum, it suddenly reacts in contact with oxygen and water to give aluminum oxide Al_2_O_3_. In addition, some elements that are part of alloy AA6061’s composition formed oxide and hydroxides in an aqueous environment, and depending on their nature, corrosion resistance is worsened or improved. Several authors have postulated the mechanism of aluminum corrosion in diluted acid solutions in a similar fashion to that occurring on iron, though considering that the aluminum surface is free from oxides; in fact, the dissolution mechanism of aluminum corrosion in acid and alkaline environments is still under discussion [50,51,52].

#### 3.5.1. Anodic Reactions

**Figure 11:**
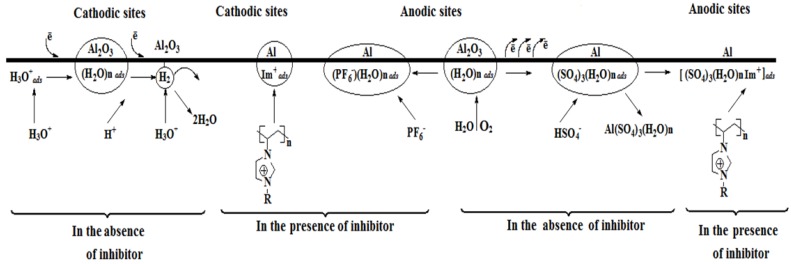
Proposed mechanism of corrosion and inhibition of aluminum alloy AA6061 before/after PIL addition in diluted sulfuric acid.

In Figure 11, corrosion and inhibition mechanisms are suggested to explain aluminum alloy behavior. In the anodic zones of alloy AA6061 in the absence of PILs, a protective oxide film is formed on the surface (Equation (8)), which can be destroyed under the action of the diluted sulfuric acid (Equation (9)) by the interaction of the hydrated film of aluminum oxide with bisulfate anions when Al_2_(SO_4_)_3_(H_2_O)*_n_*,_ads_ is formed. As this complex is soluble in the aqueous environment, it is then desorbed from the surface, leaving free active sites to be attacked again by the anions of HSO_4_^−^ or SO_4_^2−^.

4Al + *n*H_2_O + 3O_2_ → 2Al_2_O_3_(H_2_O)*_n_* (8)

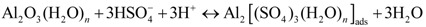
(9)

In the presence of PILs, the oxidation reactions are modified; the chemical species Al_2_[(SO_4_)_3_(H_2_O)*_n_*]_ads_ can absorb one more bisulfate anion and then interact electrostatically with the cation of each repeating unit (Im^+^) of the polymer through the imidazole ring, while the hydrophobic part of the alkylic side chain is preferentially oriented towards the solution, forming a hydrophobic barrier by the formation of Al_2_H[(SO_4_)_4_^−^(H_2_O)*_n_*Im^+^]_ads_ (Equation (10)). At the same time, the ion PF_6_^−^ can be adsorbed onto the aluminum alloy AA6061 surface, which led to an excess of negative charge (Equation (11)). The chemical species Al[(H_2_O)*_n_*(PF_6_)_4_^−^] could then electrostatically interact with the Im^+^ (Equation (12)). However, the concentration of HSO_4_^−^ in solution appeared to be higher than that of PF_6_^−^, so that the presence of Al_2_H[(SO_4_)_4_^−^ (H_2_O)*_n_* Im^+^]_ads_ is more likely.


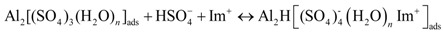
(10)

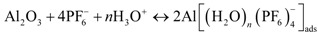
(11)

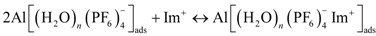
(12)

#### 3.5.2. Cathodic Reactions

As the aluminum surface is covered with oxides, which generally have dissimilar dielectric properties, it is more likely that the cathodic reactions occurred both on the metal grain boundaries and on those separating the anodic zones themselves. This way, protons coming from acid dissociation, when approaching the interior of the Helmholtz plane in the cathodic zones, are reduced to release molecular hydrogen and consume the aluminum free electrons (anodic zone) during their oxidation.


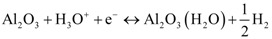
(13)

In the presence of CIs, macromolecules competed with protons to occupy active sites on the cathodic zones, which are electrostatically adsorbed through Im^+^, leading to the adsorption of AlIm_ads_ (Equation (14)). As the proton size is much smaller than that of Im^+^, the latter can cover a more extended area; so, the kinetics of the cathodic reaction is slowed and corrosion mitigated.


Al + Im^+^ + e^−^ ↔ AlIm_ads_ (14)

Some of the properties provided by the protective film on aluminum alloy AA6061 are as follows: (1) The salts of Al_2_H[(SO_4_)_4_^−^(H_2_O)*_n_* Im^+^]_ads_ and Al[(H_2_O)*_n_*(PF_6_)_4_^−^Im^+^]_ads_ adsorbed on the metallic surface could change its polarity towards the solution, which led to PF_6_^−^ and//or HSO_4_^−^ interactions that may induce a multilayer formation on the protective film that functions as a barrier against the aggressive ions in solution; (2) The steric effect of the alkylic side chain located in the imidazole ring through the entire length of the polymeric chain works favorably as hydrophobic areas; additionally, two macromolecules can form a relatively more compact polymeric film owing to opposite charges. This property contributed to the fact that PImC_12_ was derived as the most efficient of the PILs tested.

As previously described, the inhibition mechanism proposed that PILs in H_2_SO_4_ interacted on both the anodic and cathodic sites formed on the metallic surface. However, results from electrochemical tests indicated that anodic reactions predominated at the metal solution interface after long time periods; namely, at short times, the PILs affected both the anodic and cathodic reactions, but after 9 h, the kinetics of the oxidation reaction predominated due to the formation of Al_2_H[(SO_4_)_4_^−^(H_2_O)*_n_*Im^+^]_ads_.

## 4. Conclusions

From the study of poly(1-vinyl-3-alkylimidazolium hexafluorophosphate)s as corrosion inhibitors to protect aluminum alloy AA6061 in aqueous sulfuric acid, the following conclusion were derived:

(1) PILs displayed low efficiencies as corrosion inhibitors against uniform corrosion; apparently, this was derived from the highly active competition of aggressive sulfate species and inhibitor molecules to occupy the metallic surface area. ICP-OES indicated a decrease of dissolved Al^3+^ in solution after PILs, which confirmed their inhibitive action.

(2) PILs inhibitors contributed in decreasing density, morphology and depth of pitting corrosion, though not in a complete scheme, as aluminum alloy AA6061 developed pit regions in aqueous sulfuric acid after relatively long immersion times and low concentrations of PILs.

(3) Inhibitor efficiency improved with the increase in PILs concentration and the decrease in acid concentration in solution. The efficiency of these compounds as corrosion inhibitors displayed the following order: PImC_12_ > PImC_8_ > PImC_4_; in any case, *I*_E_ was shown to be time dependent.

(4) PILs adsorption on aluminum alloy in the acidic media follows Langmuir’s isotherm; *K*_ads_ values indicated that PImC_12_ is adsorbed more easily on surface than the other PILs of shorter alkylic side chains and lower molecular weights, whereas 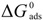 values suggested that physisorption is the process in control for inhibition.

(5) The synthesized PILs displayed a short protection range for the alloy AA6061. Thus, these PILs are not suitable to be applied in acidic media, as they are not easily adsorbed due to ionic competition, which promotes the formation of a non-uniform CI film on the aluminum alloy surface.
